# Lung Surfactant Lipids Provide Immune Protection Against *Haemophilus influenzae* Respiratory Infection

**DOI:** 10.3389/fimmu.2019.00458

**Published:** 2019-03-18

**Authors:** Belén García-Fojeda, Zoe González-Carnicero, Alba de Lorenzo, Carlos M. Minutti, Lidia de Tapia, Begoña Euba, Alba Iglesias-Ceacero, Sonia Castillo-Lluva, Junkal Garmendia, Cristina Casals

**Affiliations:** ^1^Department of Biochemistry and Molecular Biology I, Complutense University of Madrid, Madrid, Spain; ^2^Centro de Investigación Biomédica en Red de Enfermedades Respiratorias (CIBERES), Instituto de Salud Carlos III, Madrid, Spain; ^3^Instituto de Agrobiotecnología, Mutilva, Spain

**Keywords:** nontypeable *Haemophilus influenzae*, pulmonary surfactant, phospholipids, alveolar epithelial cells, host-pathogen interaction, bacterial invasion, RAC-1, PI3K/Akt

## Abstract

Non-typeable *Haemophilus influenzae* (NTHi) causes persistent respiratory infections in patients with chronic obstructive pulmonary disease (COPD), probably linked to its capacity to invade and reside within pneumocytes. In the alveolar fluid, NTHi is in contact with pulmonary surfactant, a lipoprotein complex that protects the lung against alveolar collapse and constitutes the front line of defense against inhaled pathogens and toxins. Decreased levels of surfactant phospholipids have been reported in smokers and patients with COPD. The objective of this study was to investigate the effect of surfactant phospholipids on the host-pathogen interaction between NTHi and pneumocytes. For this purpose, we used two types of surfactant lipid vesicles present in the alveolar fluid: (i) multilamellar vesicles (MLVs, > 1 μm diameter), which constitute the tensioactive material of surfactant, and (ii) small unilamellar vesicles (SUVs, 0.1 μm diameter), which are generated after inspiration/expiration cycles, and are endocytosed by pneumocytes for their degradation and/or recycling. Results indicated that extracellular pulmonary surfactant binds to NTHi, preventing NTHi self-aggregation and inhibiting adhesion of NTHi to pneumocytes and, consequently, inhibiting NTHi invasion. In contrast, endocytosed surfactant lipids, mainly via the scavenger receptor SR-BI, did not affect NTHi adhesion but inhibited NTHi invasion by blocking bacterial uptake in pneumocytes. This blockade was made possible by inhibiting Akt phosphorylation and Rac1 GTPase activation, which are signaling pathways involved in NTHi internalization. Administration of the hydrophobic fraction of lung surfactant *in vivo* accelerated bacterial clearance in a mouse model of NTHi pulmonary infection, supporting the notion that the lipid component of lung surfactant protects against NTHi infection. These results suggest that alterations in surfactant lipid levels in COPD patients may increase susceptibility to infection by this pathogen.

## Introduction

Non-typeable *Haemophilus influenzae* (NTHi) is a non-capsulated Gram-negative bacterium that has been recognized as a major causative pathogen of mucosal infections such as otitis media in children and exacerbations of chronic obstructive pulmonary disease (COPD) in adults (1–5). NTHi is a common commensal of the human nasopharynx that induces a polymicrobial disease, typically due to concurrent or predisposing respiratory viral infection (3). In the upper respiratory tract, the main pathological condition caused by NTHi is acute otitis media, with almost 60% of the cases attributable to this bacterium (1). In the lower airways, NTHi infections are highly prevalent in individuals suffering from COPD, bronchiectasis, cystic fibrosis, and pneumonia (1, 2). In particular, NTHi is a very common bacterial colonizer in the airways of COPD patients, and is the most frequently isolated bacterium in exacerbations of COPD, contributing to inflammation and disease progression (2, 5).

One of the mechanisms likely to be involved in the persistence of respiratory infections by NTHi is its capacity to invade airway epithelial cells (2, 5–7). Intracellular NTHi has been detected in epithelial cells from bronchial biopsies of patients suffering chronic bronchitis (8) and COPD (9). Intracellular invasion of lung epithelial cells enables NTHi to escape from the host immune system and to reside inside cells with high access to essential nutrients (10). Moreover, intracellular NTHi is protected from high concentrations of antibiotics, hampering clinical treatment (11). Therefore, we put forward the notion that preventing NTHi from invading lung epithelial cells is crucially important for the prophylaxis and treatment of respiratory NTHi infections.

To penetrate into airway epithelial cells, adherence of NTHi to such cells is essential, and several adhesion molecules on NTHi have been identified (12–15). They can bind either integrin receptors on the epithelial cell surface (6) or extracellular matrix proteins that interact with the epithelium (13, 15). In healthy individuals, the alveolar epithelium is exceptionally well-defended from bacterial infection through multiple mechanisms of bacterial clearance, including expression of antimicrobial peptides, lung collectins (SP-A and SP-D), and active surveillance of airway macrophages (16–18). In this study, we wondered whether the complicated network of extracellular membranes, called pulmonary surfactant, could also protect the host from NTHi adhesion and invasion.

Pulmonary surfactant is a complex lipoprotein system, exquisitely conserved across species. Surfactant is composed of 90 wt % lipids and 10 wt % proteins. Phospholipids are the major lipid component of surfactant, especially dipalmitoylphosphatidylcholine (DPPC) (19, 20). Phosphatidylglycerol (PG) represents a major unsaturated anionic component (19, 20). Four surfactant proteins form part of this material: the hydrophobic proteins SP-B and SP-C, which are inserted in surfactant membranes and are essential for surfactant biophysical function, and the soluble collectins SP-A and SP-D, which are involved in innate immune host defense (18–24). Lung surfactant is synthesized and secreted by type II pneumocytes. After secretion to the alveolar space, SP-B/SP-C and phospholipids form a multilayered surface film at the air-liquid interface that decreases alveolar surface tension on expiration, and thus prevents lung collapse and respiratory failure (19, 20). Airway instillation of surfactant is in general use for treatment of respiratory distress syndrome in preterm babies (25, 26). Replacement surfactants consist of lipid extract preparations obtained from animal bronchoalveolar fluids, containing phospholipids, mainly DPPC, and the hydrophobic proteins SP-B and SP-C (26). Interestingly, replacement surfactants also improve recovery of animal models of otitis media (27, 28). Lipoprotein structures similar to lung surfactant seems to be present in other mucosal surfaces exposed to the external environment, suggesting the importance of these lipoprotein structures.

In healthy individuals, the amount of surfactant phospholipids (particularly saturated phosphatidylcholine) is tightly regulated and does not significantly change through life (29). However, in pathological conditions, such as COPD, pulmonary fibrosis, or pneumonia, the concentration of surfactant phospholipids decreases (30–33). Whether the decrease of surfactant lipids increases susceptibility to infection by inhaled pathogens in these respiratory diseases remains mostly unaddressed.

In the present study, we tested the hypothesis that surfactant lipids may protect against NTHi infection in the lung. We found that extracellular large surfactant aggregates bind to NTHi and act as a physical barrier that inhibits adhesion of NTHi to pneumocytes and, consequently, invasion. In addition, endocytosed small lipid vesicles interfere with cytoskeletal reorganization required for bacterial entry in pneumocytes, inhibiting NTHi invasion. The protective effect of the hydrophobic fraction of pulmonary surfactant was assessed in a mouse model of NTHi infection.

## Materials and Methods

### Isolation of Lung Surfactant and Preparation of the Surfactant Hydrophobic Fraction

Pulmonary surfactant was obtained from bronchoalveolar lavages (BAL) of male Sprague Dawley rats (Envigo). Rats (~350 g) were euthanized with carbon dioxide and the cardiopulmonary block was extracted to perform BALs with 40 ml of PBS (0.2 mM EDTA). The isolation of lung surfactant experiment was reviewed and approved by the local ethics committee (both Complutense University of Madrid and Autonomous Community of Madrid), according to Directive 2010/63/EU of the European Parliament and the Spanish law RD53/2013 on protection of animals used for experimentation. The mouse lung infection assays were conducted with the approval of Animal Experimentation Committee from the Universidad Pública de Navarra and the authorization of the local government, under the same regulation as above, and following the FELASA and ARRIVE guidelines.

Large surfactant aggregates (heavy subtype surfactant) were obtained as previously described (34, 35). Briefly, BAL was centrifuged at 100,000 g for 1 h at 4°C to obtain large surfactant aggregates in the resulting pellet, which largely contains surfactant lipids and the apolipoproteins SP-A, SP-B, and SP-C. In contrast, about 80% of the total SP-D from BAL does not sediment and remains in the supernatant. The hydrophobic fraction of lung surfactant (composed of surfactant lipids, SP-B, and SP-C) was obtained by chloroform/methanol extraction as previously reported (34, 35). The organic solvent was then evaporated to dryness under a stream of nitrogen, and traces of solvent were subsequently removed by evacuation under reduced pressure overnight. Total phospholipid was determined from aliquots of the surfactant hydrophobic fraction by phosphorus analysis (36).

Multilamellar vesicles (MLVs) of the surfactant hydrophobic fraction were prepared by hydrating the dry proteolipid film in 10 mM phosphate buffered saline (138 mM NaCl, 2.7 mM KCl), pH 7.4, (PBS) and allowing them to swell for 1 h at 55° C. After vortexing, the resulting multilamellar vesicles were used for *in vitro* and *in vivo* assays. This surfactant preparation was used to test the effect of surfactant in a mouse model of NTHi infection.

### Surfactant Lipid Vesicles Preparation

Experiments were done using a synthetic mixture of surfactant phospholipids (PL), in the form of either MLVs or small unilamellar vesicles (SUVs). Surfactant vesicles were composed of dipalmitoylphosphatidylcholine (DPPC), 1-palmitoyl-2-oleoyl-phosphatidylglycerol (POPG), and palmitic acid (PA) (Avanti Polar Lipids) at weight ratios of 23:10:1.6 as previously reported (37–39). The lipid composition of these vesicles was chosen according to the following criteria: (i) a high DPPC content, which is the main phospholipid constituent of pulmonary surfactant (~50 wt.% of the total surfactant PL); (ii) the presence of an unsaturated anionic phospholipid (POPG), which forms part of lung surfactant (~8–15 wt.%); and (iii) the presence of small amounts of palmitic acid in surfactant (19, 20).

Preparation of MLVs and SUVs from surfactant lipids was carried out as described in Sáenz et al. (37, 38), and Cañadas et al. (40). The required amounts of DPPC, POPG, and PA were dissolved in chloroform/methanol (2:1 v/v). The solvent was then evaporated to dryness under a gentle stream of nitrogen. Traces of solvent were subsequently removed in a vacuum centrifuge for 2 h. In cases where the lipofilic fluorescent tracer 1,1′-Dioctadecyl-3,3,3′,3′-tetramethylindocarbocyanine perchlorate [DiIC_18_(3)] (Thermofisher Scientific) was incorporated in the lipid mixture, the lipofilic probe was dissolved in methanol and added to the lipid mixture at a DiIC_18_/surfactant phospholipid molar ratio of 1:200, before solvent removal. MLVs were prepared by hydrating the dry lipid film with PBS, allowing them to swell for 1 h at 45°C, a temperature above the gel to liquid phase transition temperature (*T*m) of these membranes (37, 38). To prepare SUVs, the resulting MLVs were sonicated at the same temperature (45°C) during 8 min at 390 W/cm^2^ (burst of 0.6 s, with 0.4 s between bursts) in a UP 200S sonifier with a 2 mm microtip (Hielscher Ultrasonics).

The size of lipid vesicles was measured at 25°C in a Zetasizer Nano S (Malvern Instruments, Malvern, UK) equipped with a 633-nm HeNe laser, as previously reported (18, 22). Three scans were performed for each sample. Zeta potential measurements were performed with a Zetasizer Nano S (Malvern Instruments), applying an electric field across the dispersion. Measurements were performed in PBS in the presence and absence of 0.4 mM Ca^2+^.

### Bacterial Strains, Media, and Growth Conditions

The two NTHi strains used in this study are clinical isolates from patients with otitis media (NTHi375) (6) and COPD (NTHi398) (14). Frozen stocks of NTHi strains were thawed and then grown on chocolate agar plates (bioMérieux) or on brain heart infusion broth (BHI) supplemented with haemin 10 μg/ml and β-NAD 10 μg/ml (Sigma-Aldrich) (sBHI). Bacteria were grown overnight at 37°C in a humidified 5% CO_2_ atmosphere.

### Epithelial Cell Cultures and Bacterial Infection

Experiments were performed using the mouse lung epithelial cell line MLE-12 (ATTC® CRL-2110^TM^, Manassas, VA, USA) and the human alveolar basal epithelial cell line A549 (ATTC® CCL-185^TM^). Cells were maintained in RPMI 1640 supplemented with 10% (v/v) heat-inactivated fetal bovine serum (FBS), antibiotics (100 U/ml penicillin and 100 μg/ml streptomycin), and 2 mM L-glutamine (BioWhittacker). Lung epithelial cells were incubated at 37°C in a humidified 5% CO_2_ atmosphere.

Bacterial adhesion and invasion assays were performed as described previously (6, 7) in the presence and absence of surfactant phospholipid vesicles (MLVs or SUVs). MLE-12 or A549 cells were seeded to a density of 50,000 cells per well in 24-well tissue culture plates for 24 h. Cells were grown in complete medium with 5% FBS [RPMI 1640 tissue culture medium supplemented with antibiotics (100 U/ml penicillin and 100 μg/ml streptomycin), 2 mM L-glutamine, and 5% FBS]. A confluence of 90% was reached at the time of the bacterial infection. The following day, cells were incubated with NTHi in the presence or absence of MLVs. For SUVs, cells were (i) pre-incubated with SUVs during 24 h to allow PL endocytosis before NTHi infection, (ii) co-incubated with SUVs at the onset of NTHi infection, or (iii) post-incubated with SUVs after NTHi infection.

For NTHi infection, bacteria were recovered with 1 ml PBS from a chocolate agar plate grown overnight. Bacterial suspensions were adjusted to OD_600_ = 1, ~10^9^ colony forming units (C.F.U.)/ml. Cells were infected with 50 μl (10^8^ C.F.U./ml) of the adjusted bacterial suspension in 1 ml of Hank's balanced buffered saline (HBBS) (137 mM NaCl, 5.4 mM KCl, 0.25 mM Na_2_HPO_4_, 0.44 mM KH_2_PO_4_, 1.3 mM CaCl_2_, 1 mM MgSO_4_, 4.2 mM NaHCO_3_).

For adhesion experiments, cells were infected for 30 min, washed three times with PBS, and lysed with 300 μl of 0.025% (w/v) saponin in PBS for 10 min at room temperature. The resulting lysates were diluted serially 1/10 in PBS, and serial dilutions were plated on sBHI agar (6, 7). Colonies were counted and the results were expressed as the percentage (%) of C.F.U. related to the control (not incubated with lipids).

For invasion assays, cells were infected for 2 h and washed three times with PBS. Cells were then incubated for an additional 1 h with RPMI 1640 containing 10% FBS and 200 μg/ml gentamicin to kill extracellular bacteria. Then, cells were washed three times with PBS and lysed as described above. Serial dilutions were plated on sBHI agar (6, 7). Colonies were counted and the results were expressed as % C.F.U. related to the control (not incubated with lipids).

In some experiments, cells were pre-incubated in the presence of anti-SR-BI blocking antibody for 30 min. Rabbit IgG anti-SR-BI [NB400-113 (Novus, Biologicals)] or its corresponding normal Rabbit IgG control (R&D Systems) were used at a working concentration of 1:100 in culture medium. Then, SUVs (250 μg/ml) were added to the medium for an additional 2 h. Afterwards, the NTHi infection assay was carried out as described above.

### Bacterial Killing Assay

NTHi strains were grown on chocolate agar. Bacteria were harvested in PBS, NaCl 100 mM, 1% (w/v) trypticasein soy broth to a final concentration of 10^8^ C.F.U./ml. Then, 100 μl of bacterial suspension (10^7^ C.F.U.) were incubated with either 250 μg/ml of PL (present as MLVs) or 20 μg/ml of polymyxin B (PMB) for 2 h at 37°C in a humidified 5% CO_2_ atmosphere. Bacteria were then stained with 5 μM 5-(and-6)-carboxyfluorescein diacetate, succinimidyl ester (CFSE) (ThermoFisher Scientific), which stains active and inactive bacteria (41). Bacteria were centrifuged, washed with PBS, and resuspended in 100 μl PBS with 7.5 μM propidium iodide, to stain dead bacteria. Bacteria were incubated for 5 min at 37°C in a humidified 5% CO_2_ atmosphere, and then centrifuged, resuspended in 10 μl PBS, and placed on a microscope slide to be immediately analyzed. Micrographs were taken with a 40x objective in a fluorescence microscope Nikon ECLIPSE TE2000-U. Dead and live bacteria were counted in six micrographs of each experimental condition using Image J software. Data are shown as % of dead bacteria to total bacteria.

### Bacterial Aggregation Assay

Otitis NTHi was grown on chocolate agar for 16 h and diluted in HBSS without CaCl_2_ to OD_600_ = 1. Bacterial aggregation was assessed by measuring changes in light absorbance at 600 nm during 3 h without shaking, in a spectrophotometer DU-800 (Beckman Coulter, Fullerton, USA). Readings were taken every 3 min. Aggregation is observed as a decrease in absorbance as bacterial aggregates precipitate out of solution. To test the effect of surfactant phospholipids (MLVs or SUVs) on this process, a suspension of NTHi was carefully mixed with and without surfactant lipids (MLVs or SUVs) at room temperature, and bacterial aggregation was measured. Control experiments with phospholipid vesicles alone, without bacteria, were performed.

### Confocal Microscopy

Endocytosis of lipid vesicles (either MLVs or SUVs) were analyzed by confocal microscopy. Cells were seeded to a density of 50,000 cells per well in 24-well tissue culture plates for 24 h. Each well-contained a plastic coverslip previously sterilized under UV light for 10 min. Cells were grown in complete medium with 5% FBS. The following day, cells were incubated for 5, 10, 30 min, 1, 4, or 24 h with MLVs or SUVs (250 μg PL/ml), fluorescently labeled with DiIC_18_(3) (λ_exc_ = 549 nm; λ_em_ = 565 nm).

To determine whether the internalization of PL was mediated by the scavenger receptors SR-BI or CD36, confocal microscopy experiments were performed in the presence of either blocking antibodies [mouse IgAκ anti-CD36 (Abcam) and rabbit IgG anti-SR-BI] or their isotype controls [mouse IgAκ isotype control (Abcam) and normal rabbit IgG control], at a working concentration of 1:100, for 30 min. Then, cells were incubated for 1 h with lipid vesicles (250 μg PL/ml) stained with DiIC_18_(3).

After incubation with vesicles, cells were washed three times with PBS and fixed with 4% paraformaldehyde (PFA) in PBS for 30 min at room temperature. Staining of plasma membrane and organelles was performed with 5 μg/ml wheat germ agglutinin (WGA) conjugated with Alexa Fluor 488 for 10 min at room temperature. Nuclei were stained with 1 μg/ml 4′, 6-diamino-2-phenylindol (DAPI) for 5 min. Coverslips were mounted onto glass slides using ProLong Diamond (Thermo Fisher Scientific), and micrographs were taken under an Olympus FV1200 confocal system.

NTHi invasion of alveolar epithelial cells was also analyzed by confocal microscopy as in Morey et al. (6) and López-Gómez et al. (7). Once invasion experiments were performed and cells were treated with gentamicin, as described above, cells were washed three times with PBS and fixed with 4% PFA in PBS for 30 min at room temperature. Then cells were permeabilized with 0.1% saponin in PBS, and staining was performed in 10% FBS and 0.1% saponin in PBS. NTHi was stained with rabbit anti-NTHi antibody at a working concentration of 1:800 (6), followed by a secondary antibody conjugated with Alexa Fluor 488 (1:100). Late endosomes were stained with anti-LAMP-1 antibody conjugated with phycoerythrin at a working concentration of 1:300 (eBioscience). Nuclei were stained with 1 μg/ml DAPI for 5 min. Coverslips were mounted onto glass slides using ProLong Diamond, and images were taken with an Olympus FV1200 confocal system. Quantification of internalized NTHi bacteria (colocalized with late endosomes) per cell number was performed using Image J software on each of 12 micrographs per treatment and experiment.

### Flow Cytometry

Alveolar epithelial cells were seeded to a density of 80,000 cells per well in 24-well tissue culture plates and were grown overnight in complete medium with 5% FBS. For analysis of endocytosis of SUVs, cells were incubated for 30 min with or without either blocking antibodies for scavenger receptors (mouse IgAκ anti-CD36 and rabbit IgG anti-SR-BI) or their appropriate isotype controls, all used at a working concentration of 1:100 in medium. Then, SUVs fluorescently labeled with DiIC_18_(3) were added to the cells at a concentration of 100 μg PL/ml, and cells were incubated with lipid vesicles for the indicated times. Next, cells were trypsinized and collected in tubes for centrifugation. After two washes with PBS, cells were resuspended in 200 μl PBS and analyzed by flow cytometry using Becton-Dickinson FACSCan and Cell Quest software.

To test the effect of phospholipid uptake on the expression of SR-BI or CD36 receptors on the cell surface, cells were incubated in the presence or absence of SUVs (250 μg PL/ml) for 2 h. Then cells were harvested with cold PBS 10 mM EDTA and incubated with 10% FBS-supplemented medium, followed by incubation with either anti-SR-BI, anti-CD36, or isotype controls and appropriate secondary Alexa Fluor 488-conjugated antibodies. Following surface staining, samples were analyzed by flow cytometry using Becton-Dickinson FACScalibur and Cell Quest Pro software.

### Western Blot Analysis of Akt Phosphorylation

Cells were seeded to a density of 50,000 cells per well in 24-well tissue culture plates and were grown overnight in complete medium with 5% FBS. The following day, cells were pre-incubated for 24 h with different concentrations of surfactant PL (present in solution as SUVs) to allow their endocytosis by alveolar epithelial cells. Then, cells were infected with NTHi following the same conditions previously explained for invasion experiments. After 45 min of cell-pathogen contact, cells were washed three times with PBS and lysed with 120 μl lysis buffer composed of 10 mM HEPES, 1.5 mM MgCl_2_, 10 mM KCl, 0.5 mM EDTA, 0.2% Triton X-100, 1 mM benzamidine, 20 μg/ml aprotinin, 20 μg/ml leupeptin, 20 mM β-glycerophosphate, 10 mM NaF, 10 mM sodium pyrophosphate, and 2 mM orthovanadate (Sigma-Aldrich). Samples were resolved by SDS-PAGE under reducing conditions and transferred to polyvinylidene fluoride (PVDF) membranes (Bio-Rad). Membranes were blocked with 5 % (w/v) skimmed milk in PBS and were incubated with the anti-phospho-Akt (Ser473) antibody (1:5000), or Akt antibody (Cell Signaling) (1:1000), overnight at 4°C. After a washing step in PBS, membranes were incubated for 1 h at room temperature with the appropriate secondary antibody conjugated with horseradish peroxidase (Cell Signaling). Membranes were washed in PBS and exposed to ECL reagents (Millipore). Immunoreactive bands were quantified using Quantity One Software (Bio-Rad). Results are shown as p-Akt normalized to total Akt, and expressed as % of pAkt/Akt induced by NTHi in cells in the absence of surfactant PL. Data were presented as the p-Akt intensity of each band normalized for the intensity of the corresponding total Akt band (same sample) and referred as the correspondent percentage in relation to cells infected with NTHi but not treated with phospholipids.

### Rac1-GTP Pull Down

Pull down assays were performed as described in Castillo-Lluva et al. (42) and Woodcock et al. (43). Cells were seeded to a density of 500,000 cells per plate in 60 cm diameter tissue culture plates and were grown in complete medium with 5% FBS. The following day, SUVs (250 μg PL/ml) were added to the cells for 24 h to allow PL endocytosis. Then cells were infected with NTHi for 2 h as described above for bacterial invasion experiments.

To measure endogenous Rac GTPase activity, cells were lysed on ice in glutathione-S-transferase-fish buffer (50 mM TrisHCl, pH 7.5, 100 mM NaCl, 2 mM MgCl2, 1% (v/v) Nonidet P-40, 10% (v/v) glycerol, and protease inhibitors [0.5 mM benzamidine, 10 μg/ml aprotinin, 10 μg/ml leupeptin] (Sigma-Aldrich) containing 1.5 μg of a biotinylated p21-activated kinase (PAK) derived CRIB (Cdc42/Rac interacting binding) peptide per assay. Cleared cell lysates were incubated at 4°C for 30 min. Active Rac/CRIB complexes were precipitated using streptavidin-conjugated magnetic sepharose beads (GE Healthcare) for a further 15 min at 4°C. Following washes in glutathione-S-transferase-fish buffer, protein samples were retrieved with 1× SDS-PAGE sample buffer and processed for Western blotting with anti-Rac antibody (Becton Dickinson).

### NTHi Mouse Lung Infection

CD1® IGS (Caesarian-derived 1 from Charles River Laboratories, Massachusetts, U.S.A.) mice were used to establish a model of NTHi lung infection, as previously described (44, 45). CD1 female mice (18–20 g) aged 4–5 weeks were purchased from Charles River Laboratories (France), housed under pathogen-free conditions at the Institute of Agrobiotechnology facility (registration number ES/31-2016-000002-CR-SU-US), and used at 22–25 g. Before NTHi infection, the surfactant hydrophobic fraction (or the same volume of PBS in the untreated group) was intratracheally instilled into the lung at 37°C at a dose of 25 mg PL/kg body weight (40 μl of surfactant at a PL concentration of 13.75 mg/ml). Subsequently, 10 μl of PBS (uninfected group) or a NTHi375 suspension containing 4 × 10^9^ C.F.U./ml (4 × 10^7^ C.F.U./mouse) (NTHi infected group) were intratracheally administered. Mice were randomly divided into 6 infected (*n* = 5) groups and 2 non-infected (*n* = 3) control groups. The NTHi-infected groups are: (i) animals treated with surfactant, euthanized at 6 h post-infection; (ii) treated with surfactant, euthanized at 12 h post-infection; (iii) treated with surfactant, euthanized at 24 h post-infection; and untreated groups instilled with PBS, (iv) euthanized at 6 h, (v) 12 h, and (vi) 24 h post-infection. The uninfected control groups are: (1) animals treated with surfactant; and (2) animals instilled with PBS.

In all animals, lungs were aseptically removed and weighed. The left lung was clamped and separated to obtain lung homogenates in 1:10 (w/v) sterile PBS. The right lung was lavaged five times with sterile PBS to obtain the bronchoalveolar lavage (BAL). BAL fluid was centrifuged at 2000 × g for 10 min, and the pellet was resuspended in 1 ml RPMI 10% FBS as described previously (18). A 50-μl aliquot was stained with an equal volume of 0.4% trypan blue (Sigma-Aldrich) for total cell count on a hemocytometer. Differential cell counts were made on cytospin preparations stained with May–Grünwald–Giemsa (Sigma-Aldrich). For bacterial count in cell-free BAL and lung homogenate, dilutions of BAL and lung homogenates were plated in triplicate on BHI-agar plates, and the number of CFUs was determined after an overnight incubation at 37°C. Data were expressed as log_10_ CFU ± SEM/mouse.

### Statistical Methods

Data are presented as means ± SEM. Differences in means between groups were evaluated by one-way ANOVA, followed by the Bonferroni multiple comparison test. Student's *t*-test was used when comparing two groups. An α level ≤ 5% (*p* ≤ 0.05) was considered significant. SigmaPlot version 11.0 was used for statistical tests.

## Results

Two different surfactant subtypes are present in the alveolar fluid: (i) a heavy subtype or large surfactant aggregates, rich in SP-B and SP-C, that form MLVs in solution and constitute the freshly secreted surfactant tensioactive material; and (ii) a light subtype characterized by the presence of small vesicles devoid of SP-B and SP-C and by poor surface activity. These small vesicles are generated after inspiration/expiration cycles (19).

In this study, we used two types of lipid vesicles with different sizes, composed of either the hydrophobic fraction of surfactant (PL/SP-B/SP-C) or a mixture of surfactant lipids (DPPC/POPG/PA). Table 1 shows the average size distribution and zeta potential of MLVs and SUVs used in this study, determined in PBS in the absence and presence of physiological concentrations Ca^2+^. Zeta potential measurements of lipid vesicles used in this study confirm that they are negatively charged due to the presence of anionic phospholipids. The Zeta potential value for lipid vesicles composed of a surfactant lipid mixture was −39.5 ± 0.6 mV, similar to that obtained for the hydrophobic fraction of native surfactant: −43.8 ± 0.1 mV (Table 1). The presence of calcium decreased Zeta potential values to −27.5 ± 0.3 and −30.4 ± 0.3 mV, respectively.

**Table 1:** The average size distribution and zeta potential of MLVs and SUVs used in this study, determined in PBS in the absence and presence of physiological concentrations Ca^2+^.

**Vesicle type**	**Composition**	**[Ca^**2+**^] (mM)**	**Z-average (nm)**	**Z-potential (mV)**
MLVs	PL/SP-B/SP-C (Native surfactant)	0.0	2,651 ± 45	−43.8 ± 0.1
		0.4	2,380 ± 28	−30.4 ± 0.3
Downsizing MLVs (sonication) **(a)**	PL/SP-B/SP-C (Native surfactant)	0.0	283 ± 2	−39.0 ± 0.2
		0.4	648 ± 15	−24.0 ± 0.2
MLVs	DPPC/POPC/PA (Surfactant lipid mixture)	0.0	1,057 ± 17	−39.5 ± 0.6
		0.4	3,100 ± 27	−27.5 ± 0.3
Downsizing MLVs (sonication) **(b)** SUVs	DPPC/POPC/PA (Surfactant lipid mixture)	0.0	98 ± 5	−36.0 ± 0.3
		0.4	110 ± 8	−26.0 ± 0.4

*Phospholipid (PL) concentration: 0.25 mg/mL*.

(a) *Small vesicles from the hydrophobic fraction of native surfactant were not used in this study due to their instability and rapid aggregation induced by the presence of surfactant hydrophobic proteins SP-B and SP-C. They aggregated in the presence of calcium and tend to rapidly aggregate over time*.

(b) *This sample showed a polydispersed size-distribution, with two major peaks centered at 60 ±7 nm and 31 ± 2 nm, and a minor peak at 677 ± 5. The average size is ≤ 100 nm (SUVs)*.

MLVs used in this study mimic the lung surfactant multilayers present in the hypophase of the air-liquid interface in the alveolus, whereas SUVs resemble the small lipid vesicles produced as a consequence of breathing compression-expansion cycles.

### Lipid Vesicle Uptake by Alveolar Epithelial Cells Depends on Vesicle Size

Alveolar epithelial cells are always in contact with surfactant lipid vesicles and ingest abundant amounts of this material to maintain surfactant homeostasis. However, the process of lipid uptake by alveolar epithelial cells and the receptors involved are incompletely understood.

**Figure 1:**
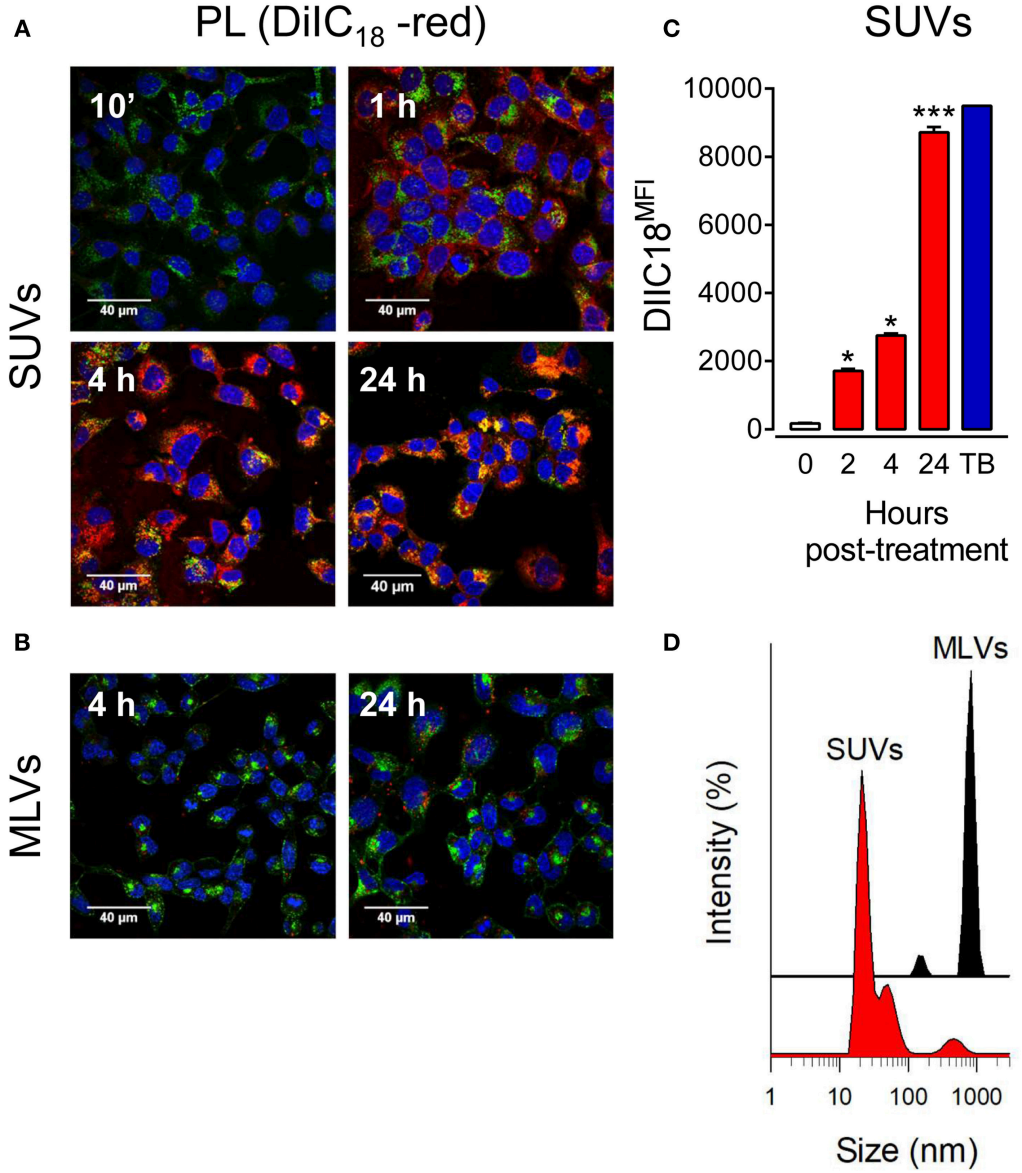
Small vesicles, but not multilamellar vesicles, of pulmonary surfactant are internalized by pneumocytes. **(A)** MLE-12 pneumocytes were incubated with DiIC_18_(3)-labeled SUVs composed of a mixture of surfactant lipids (250 μg PL/ml) for 10 min, 1, 4, or 24 h. Cells were fixed and stained with Alexa Fluor 488-conjugated WGA. Nuclei were stained with DAPI. Endocytosis of lipid vesicles was analyzed by confocal microscopy. **(B)** As in **(A)**, but cells were incubated with DiIC_18_(3)-labeled MLVs (250 μg PL/ml) for 4 and 24 h. **(C)** Cells were incubated with DiIC_18_(3)-labeled SUVs for the indicated times and analyzed by flow cytometry. ANOVA followed by Bonferroni multiple comparison test was used. ^*^p < 0.05 and ^***^p < 0.001 when compared to control cells in the absence of lipids. Trypan blue (TB) was used to quench extracellular vesicles attached to the cell membrane after 24 h incubation. **(D)** Hydrodynamic diameter of surfactant vesicles (SUVs and MLVs) determined by dynamic light scattering. The y-axis represents the relative intensity of the scattered light; the x-axis denotes the hydrodynamic diameter of the particles present in the solution. One representative experiment of three is shown.

To directly assess the effect of vesicle size on lipid uptake by alveolar epithelial cells, we incubated lung epithelial cell lines (mouse MLE-12 and human A549 cells) stained with Alexa Fluor 488-conjugated wheat germ agglutinin and DAPI in the presence and absence of fluorescent lipid vesicles of different sizes (MLVs or SUVs) composed of surfactant lipids (DPPC/POPG/PA). Lipid uptake was analyzed by confocal microscopy. We observed a time-dependent increase in the engulfment of SUVs, but not MLVs, by mouse MLE-12 (Figures 1A,B) and human A549 (Supplementary Figure 1) cells. Fluorescent small lipid vesicles were observed inside pneumocytes after 1 h of incubation, and the greatest uptake of SUVs seems to occur at 24h (Figure 1A). MLVs were not internalized by mouse and human pneumocytes after 24 h (Figure 1B and Supplementary Figure 1). Flow cytometry analysis of the time-dependent entry of fluorescent small lipid vesicles in mouse pneumocytes indicated that maximal cell fluorescence was observed at 24 h (Figure 1C). At this time, no extracellular vesicles attached to the cell membrane are quenched by trypan blue. Figure 1D show the different hydrodynamic sizes of the vesicles (MLVs and SUVs) used in this study, determined by dynamic light scattering.

According to these results, MLVs, which cannot be internalized by pneumocytes, were used in this study to determine the extracellular effect of lung surfactant on NTHi infection *in vitro*, whereas SUVs, which can be internalized, can be used to analyze the intracellular effect of endocytosed surfactant lipids.

### Extracellular Large Surfactant Aggregates Bind to NTHi and Inhibit Bacterial Adhesion to Pneumocytes

**Figure 2:**
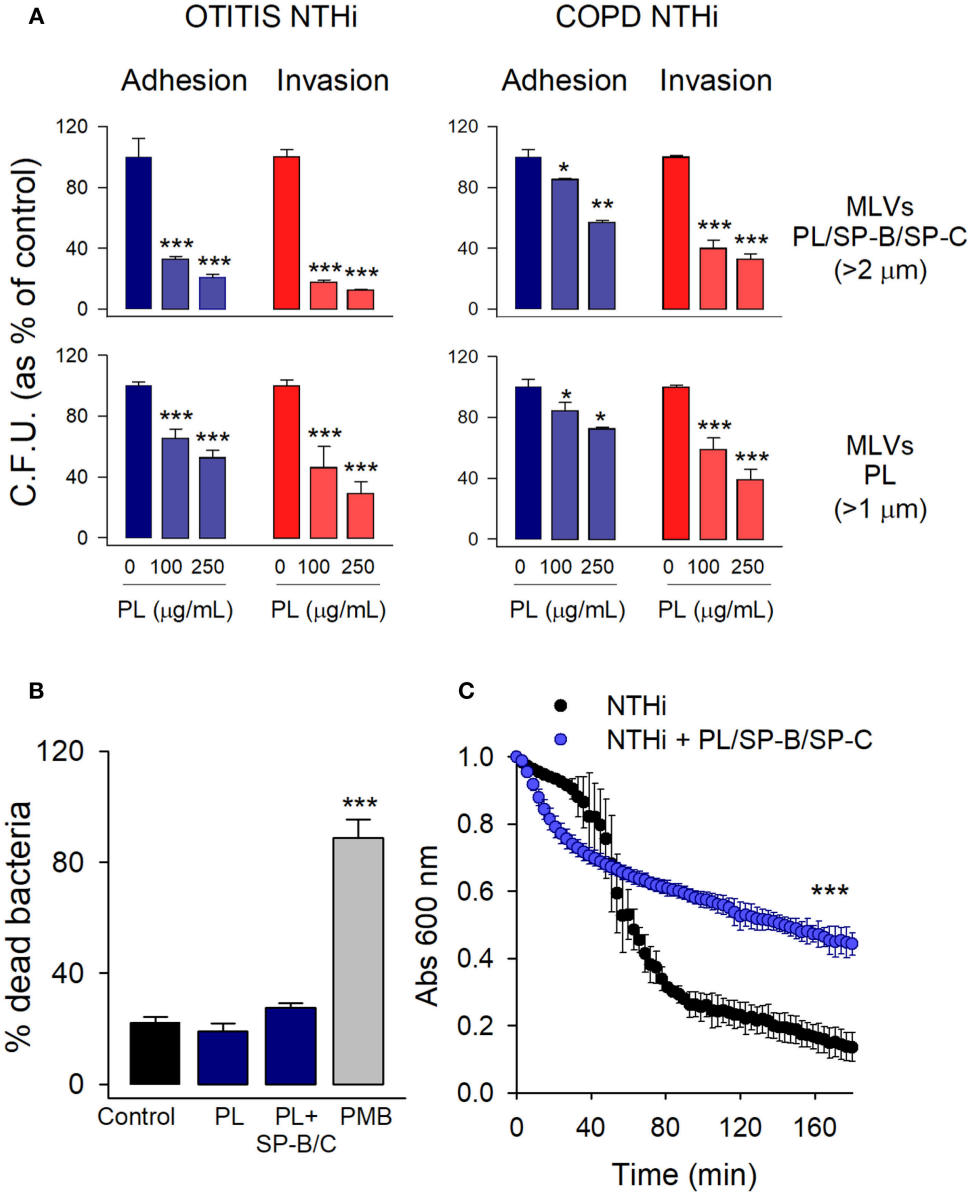
Extracellular multilamellar vesicles of pulmonary surfactant inhibit adhesion of NTHi to pneumocytes and decrease NTHi self-aggregation. **(A)** MLE-12 cells were infected with NTHi clinical strains from patients with otitis media and COPD, in the presence or absence of MLVs (100 or 250 μg PL/ml) prepared from either the hydrophobic fraction of native surfactant (PL/SP-B/SP-C) or a mixture of surfactant lipids (PL). For adhesion experiments, cells were infected for 30 min, washed, and lysed. For invasion assays, cells were infected for 2 h, washed, and incubated for an additional 1 h with gentamicin to kill extracellular bacteria. The resulting lysates were plated on sBHI agar. Colonies were counted and the results were expressed as percentages of C.F.U. relative to infected cells in the absence of lipids. Results are mean ± SEM of three independent experiments run in triplicate. ANOVA followed by Bonferroni multiple comparison test was used. ^*^p < 0.05, ^**^p < 0.01, and ^***^p < 0.001 when compared with NTHi-infected pneumocytes in the absence of lipids. **(B)** NTHi (otitis strain) were incubated in the presence or absence of MLVs (250 μg/ml) composed of either a mixture of surfactant lipids (PL), or the hydrophobic fraction of native surfactant (PL/SP-B/C) for 30 min. Bacteria were then stained with carboxyfluorescein diacetate succinimidyl ester (green), and bacterial viability was assessed by propidium iodide exclusion. Polymixin B (PMB) (20 μg/ml) was used as a positive control of bacterial killing. Data were expressed as % of dead bacteria. Results are mean ± SEM. ANOVA followed by the Bonferroni multiple-comparison test was used. ^***^p < 0.001 vs. untreated bacteria. **(C)** Effect of surfactant vesicles on NTHi self-aggregation. NTHi (otitis strain) was incubated in the presence or absence of MLVs (250 μg PL/ml) prepared from the hydrophobic fraction of native surfactant. Bacterial aggregation was monitored by measuring the decrease of absorbance at 600 nm every 3 min. Four independent experiments were performed. Results are mean ± SEM. Student's *t*-test was used. ^***^p < 0.001.

The analysis of the extracellular effect of surfactant lipids on the adhesion and invasion of NTHi to pneumocytes was performed with MLVs prepared from either the hydrophobic fraction of native surfactant (PL/SP-B/SP-C) or a mixture of surfactant lipids (PL). For adhesion experiments, cells were infected for 30 min, washed, and lysed. For invasion assays, cells were infected for 2 h, washed, and incubated for an additional 1 h with gentamicin to kill extracellular bacteria (6, 7). We used two NTHi clinical strains, isolated from patients with otitis media and COPD. Figure 2A shows that co-incubation of mouse MLE-12 cells with MLVs and NTHi strains results in reducing the number of bacteria adhered and internalized in the cells. For both NTHi strains, inhibition of bacterial adhesion and invasion was dose-dependent. For each concentration of PL, similar inhibition values were reached for adhesion and invasion of NTHi. These results suggest that the observed reduction in bacterial internalization relates to the inhibition of bacterial adhesion to the cell membrane.

To test the possibility that surfactant MLVs would have a direct bactericidal effect on NTHi that would lead to the observed decreased C.F.U. in infection experiments, we analyzed bacterial viability in the presence and absence of MLVs at the greatest PL concentration assayed. Polymyxin B was used as a positive control of bacterial killing. Bacterial viability was determined by propidium iodide exclusion. Figure 2B shows that MLVs from native or synthetic surfactant preparations did not have bactericidal action against NTHi.

To test whether NTHi can stick to MLVs, so that surfactant lipids might serve as a sink absorbing the bacteria, we analyzed NTHi aggregation in the presence and absence of MLVs prepared from the surfactant hydrophobic fraction (PL/SP-B/SP-C). Figure 2C shows that MLVs significantly decreased bacterial aggregation over time. These results indicate that surfactant MLVs bind to bacteria, impairing bacterial self-aggregation and bacterial attachment to epithelial cells.

Altogether, our results indicate that extracellular surfactant membranes would function as biological barriers that bind to bacteria and prevent bacterial adhesion to the epithelium.

### Endocytosed Surfactant Lipids Inhibited NTHi Invasion by Blocking Bacterial Uptake in Pneumocytes

**Figure 3:**
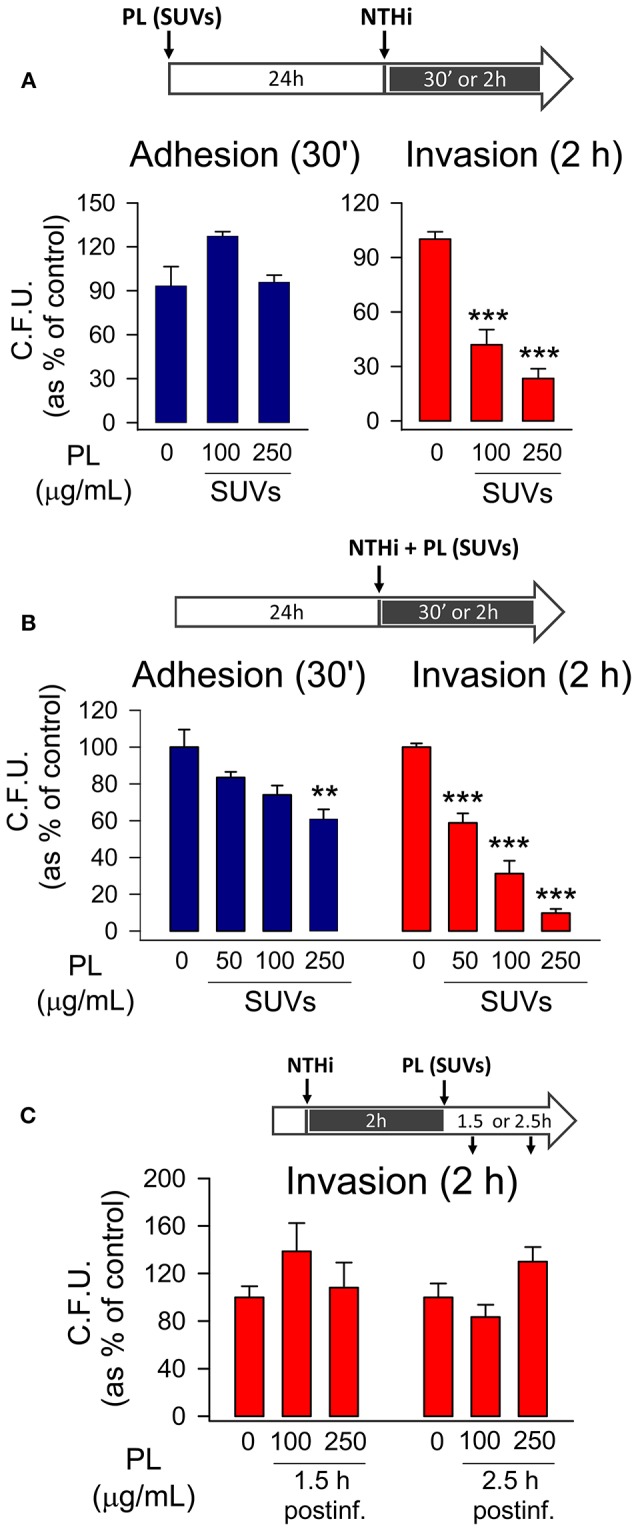
Endocytosed surfactant vesicles inhibit NTHi invasion of pneumocytes. MLE-12 pneumocytes were incubated with SUVs composed of a mixture of surfactant lipids. Cells were either pre-incubated with SUVs for 24 h prior to infection **(A)**, co-incubated with SUVs and NTHi **(B)**, or post-incubated with SUVs for 1.5 or 2.5 h after infection with NTHi **(C)**. For bacterial adhesion experiments, cells were infected for 30 min. For invasion assays, cells were infected for 2 h, washed, and incubated with gentamicin to kill extracellular bacteria. Cells were lysed and plated on sBHI agar. Data are shown as percentage of C.F.U. relative to infected cells in the absence of lipid vesicles. Results are mean ± SEM of three independent experiments performed in triplicate. ANOVA followed by the Bonferroni multiple-comparison test was used. ^**^p < 0.01, and ^***^p < 0.001 when compared with infected pneumocytes in the absence of lipids.

With the aim of analyzing intracellular effect of endocytosed surfactant vesicles on NTHi adhesion and invasion, mouse pneumocytes were preincubated with SUVs 24 h before infection. Then, cells were washed, independently infected with the two NTHi clinical strains, and both adhesion and invasion experiments were performed. Results indicated that endocytosed surfactant vesicles significantly inhibited otitis-NTHi invasion but not bacterial adhesion to mouse MLE-12 (Figure 3A) and human A549 (Supplementary Figure 2A) pneumocytes. Higher concentrations of PL are needed to inhibit COPD NTHi strain (Supplementary Figure 2B). This strain had a greater invasion capacity, as long as the number of C.F.U. recovered in invasion experiments was one order of magnitude greater than that of the otitis NTHi strain (data not shown). These experiments suggest that pneumocytes that endocytose small surfactant vesicles, as part of the surfactant recycling cycle, show reduced NTHi invasion.

When mouse pneumocytes were co-incubated with NTHi and SUVs for just 30 min, small PL vesicles also reduced bacterial adhesion to the epithelium (Figure 3B), but the PL concentration required for bacterial adhesion inhibition was greater for SUVs than for MLVs (Figure 3A). Small PL vesicles bound to NTHi decreasing NTHi self-aggregation at the highest concentration tested (Supplementary Figure 2C). Importantly, co-incubation of mouse pneumocytes with NTHi and SUVs for 2 h resulted in a robust inhibition of bacterial invasion at very low PL concentrations, likely due to SUV's endocytosis (Figure 3B). However, the effect of SUVs on NTHi invasion in coincubation experiments was greater than that observed when SUVs were previously endocytosed by epithelial cells (Figure 3A), suggesting that the effect of SUVs on reducing NTHi aggregation and bacterial adhesion might also influence bacterial invasion.

Once epithelial cells were infected by NTHi, post-infection treatment with SUVs had no effect on the number of CFU recovered in invasion experiments (Figure 3C). This suggests that uptake of SUVs post-infection did not affect survival of previously internalized bacteria.

**Figure 4:**
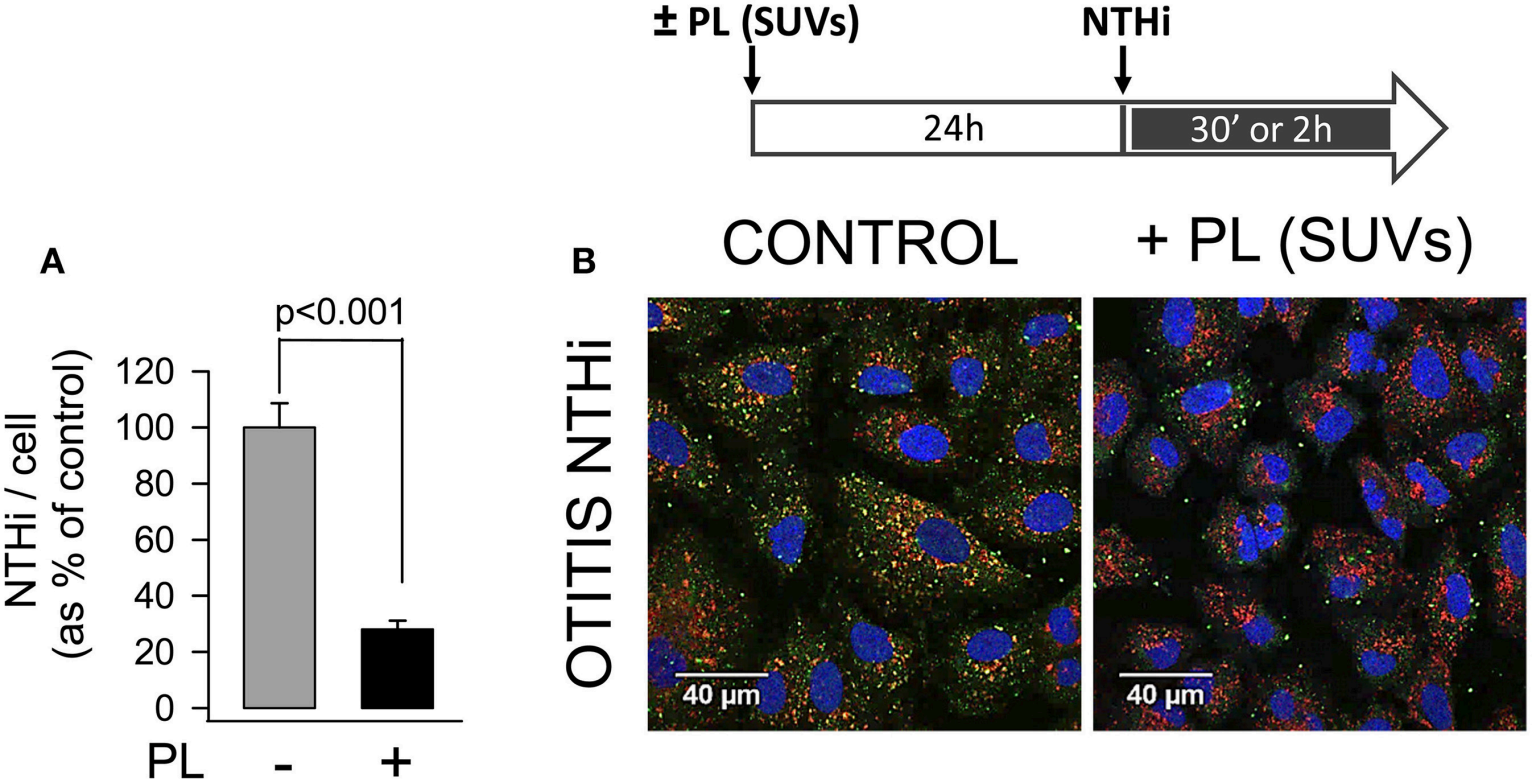
Endocytosis of surfactant lipids attenuates NTHi entry in pneumocytes. MLE-12 cells were incubated with or without SUVs composed of surfactant lipids (250 μg PL/ml) for 24 h. Then cells were infected with otitis NTHi, and bacterial invasion assays were performed. Cells were then washed, fixed, and permeabilized with 0.1% saponin. For confocal microscopy analysis, internalized bacteria were stained with an anti-NTHi antibody and Alexa Fluor 488-conjugated secondary antibody. Late endosomes were stained with anti-LAMP1 antibody conjugated with phycoerytrin, and nuclei were stained with DAPI. **(A)** The graph shows the percentage of internalized bacteria relative to cell associated individual bacteria. Mean ± SEM of two independent experiments are shown. Student's *t*-test was used. **(B)** Representative micrographs are shown.

To assess that endocytosed surfactant lipids inhibited NTHi invasion by blocking bacterial uptake in pneumocytes, we analyzed internalized fluorescent bacteria by confocal microscopy (Figure 4). To this end, bacteria were stained with an antibody against NTHi and Alexa-488-conjugated secondary antibody, late endosomes were stained with a PE-conjugated anti-LAMP1 antibody, and cell nuclei were stained with DAPI. In absence of lipids, fluorescent NTHi bacteria co-localized with late endosomes, as previously described (6). However, when cells were preincubated with small surfactant vesicles for 24 h prior to infection, a 70% reduction of fluorescent bacteria was observed inside the cells. These results indicate that endocytosed surfactant lipids inhibited NTHi entry in pneumocytes.

### Blocking Scavenger Receptor SR-BI Abrogates Surfactant Lipid Inhibition of NTHi Invasion

**Figure 5:**
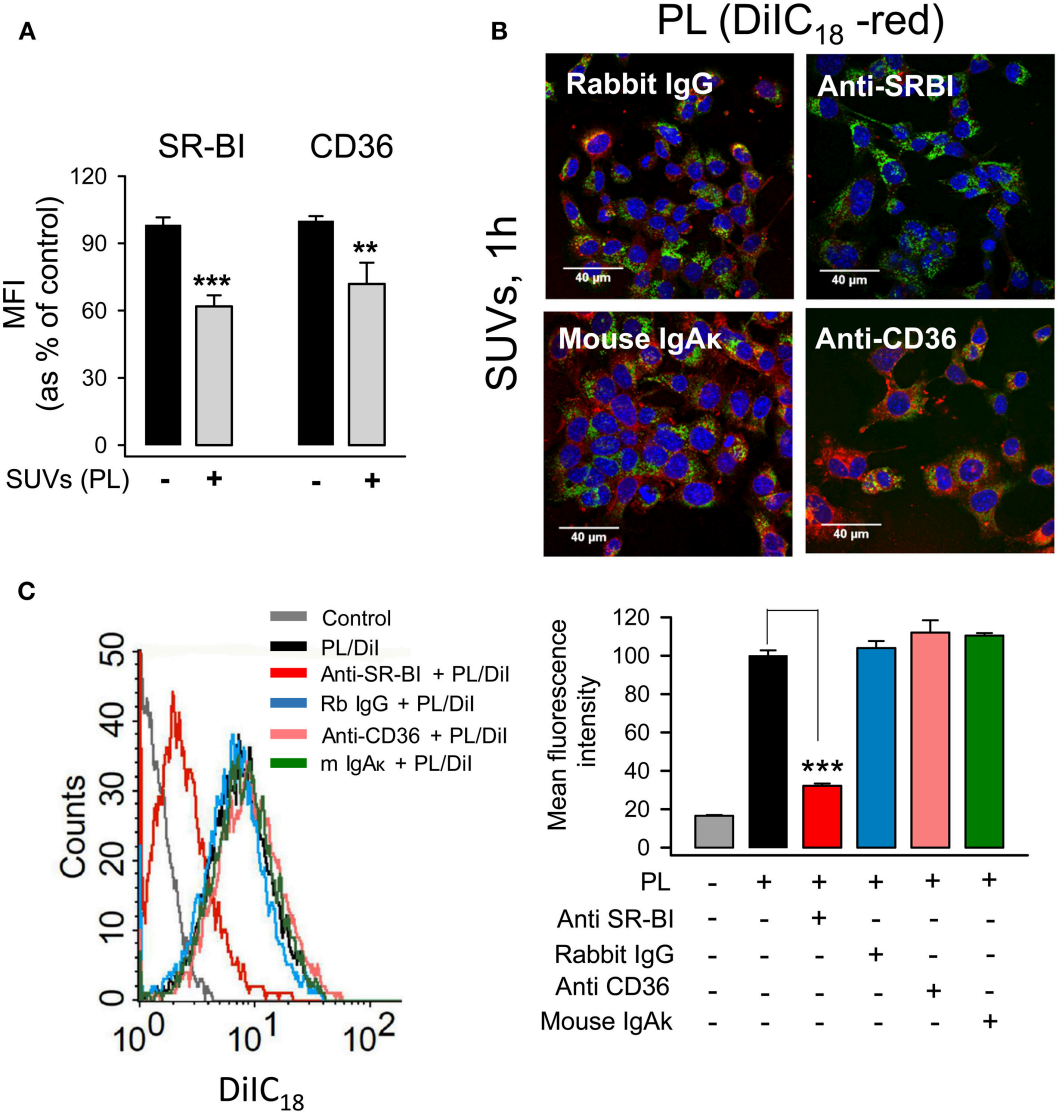
SR-BI mediates endocytosis of small surfactant vesicles by pneumocytes. **(A)** MLE-12 cells were incubated in the presence or absence of SUVs (250 μg PL/ml) for 2 h, then cells were harvested and stained with anti-SR-BI, anti-CD36 or their respective controls, rabbit IgG or mouse IgAκ, and appropriate Alexa Fluor 488-conjugated secondary antibodies. The mean fluorescence intensity (MFI) of SR-BI and CD36 was measured by flow cytometry, and background fluorescence from IgG and IgAκ controls was subtracted. Data are presented as mean ± SEM of two independent experiments run in triplicate. Student's *t*-test was used. ^**^p < 0.01, and ^***^p < 0.001 when compared with control cells in the absence of lipids. In **(B,C)**, anti-SR-BI and anti-CD36 blocking antibodies or their respective controls, rabbit IgG or mouse IgAκ, were added to MLE-12 cells for 30 min. Then cells were incubated with DiIC_18_(3)-labeled SUVs (100 μg/ml) for an additional 60 min. **(B)** Cells were fixed, stained with Alexa Fluor 488-WGA (cell membranes) and DAPI (nuclei), and analyzed by confocal microscopy. **(C)** Cells were fixed and analyzed by flow cytometry. A representative histogram is shown. The mean fluorescence intensity is represented as percentage of positive control, which is cells incubated with DiIC_18_(3)-labeled SUVs in the absence of antibodies. Data are presented as mean ± SEM of three independent experiments run in triplicate. ANOVA followed by the Bonferroni multiple-comparison test was used. ^***^p < 0.001 when compared with cells incubated with SUVs in the absence of antibodies.

Although surfactant lipids may be endocytosed by type II pneumocytes via clathrin-dependent and -independent mechanisms (46), the receptors that mediate lipid vesicle endocytosis by pneumocytes have not been entirely identified. Scavenger receptors (SR) are involved in lipid uptake (47) and SR-BI and SR-BII (CD36) interact with anionic phospholipids (48). In addition, these receptors are expressed in airway epithelial cells (49). The expression of SR-BI and CD36 receptors on the surface of MLE-12 epithelial cells was examined in the presence and absence of surfactant PL. We observed that the expression of both SR-BI and CD36 was significantly reduced in response to lipid vesicle internalization (Figure 5A).

To determine the effect of blocking antibodies of SR-BI and CD36 receptors on the endocytosis of small surfactant vesicles, alveolar epithelial cells were preincubated with blocking antibodies or their respective immunoglobulin controls, and endocytosis of DiIC_18_-fluorescent lipid vesicles was determined by confocal microscopy (Figure 5B) and flow cytometry (Figure 5C). We identified SR-BI as the receptor with a prominent role in the endocytosis of surfactant lipids, since the anti-SR-BI blocking antibody caused 70 % reduction of SUVs endocytosis by mouse and human pneumocytes (Figure 5C and Supplementary Figure 3). In contrast, anti-CD36 produced only 20 % PL endocytosis reduction by human cells, and it had no effect on PL endocytosis by mouse cells (Figure 5C and Supplementary Figure 3).

**Figure 6:**
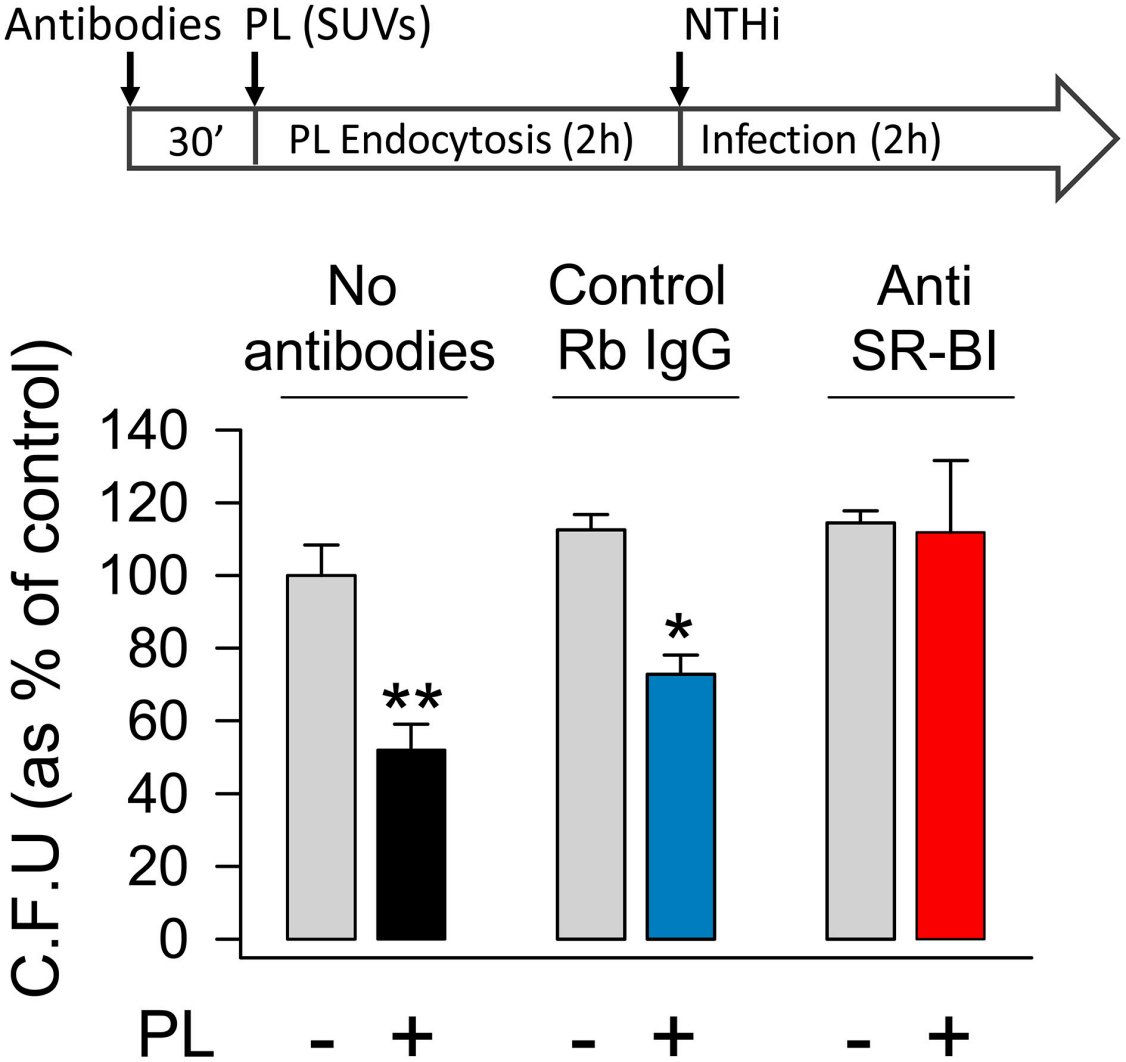
Blocking scavenger receptor SR-BI abrogates surfactant lipid inhibition of NTHi invasion. Mouse pneumocytes were preincubated with or without anti-SR-BI or control rabbit IgG antibodies for 30 min, followed by 2 h incubation with SUVs composed of a mixture of surfactant lipids (100 μg PL/ml). Then, cells were infected with otitis NTHi, and invasion experiments were performed. Bacterial invasion was quantified by colony counting, and results were expressed as percentage of C.F.U. relative to cells infected in the absence of lipids and antibodies. The percentages of C.F.U. obtained in cells infected in the absence of lipids were not affected by the presence or absence of antibodies (control IgG or anti-SR-BI). Mean ± SEM of two independent experiments done in duplicate are shown. ANOVA followed by the Bonferroni multiple-comparison test was used. ^*^p < 0.05 and ^**^p < 0.01 when compared with infected pneumocytes in the absence of surfactant lipids and antibodies.

Next, we investigated whether the engulfment of surfactant lipids by SR-BI might be critical for the attenuation of NTHi invasion (Figure 6). To this end, MLE-12 cells were pre-incubated in the presence or absence of anti-SR-BI blocking antibody or its IgG control for 30 min before incubating with surfactant lipid vesicles for 2 h. After washing, cells were infected with NTHi and bacterial invasion experiments were performed. We found that, in the absence of lipids, anti-SR-BI antibody did not have any effect on NTHi invasion, because the number of bacteria internalized in the cells was similar to that of control experiments without blocking SR-BI antibody or in the presence of its IgG control (Figure 6). Importantly, in the presence of small lipid vesicles, the blockade of SR-BI impeded lipid-induced inhibition of NTHi invasion (Figure 6). Thus, blocking the endocytosis of lipid vesicles increased pneumocyte susceptibility to NTHi invasion. These results demonstrate that SR-BI-mediated internalization of surfactant lipids is required for lipid-dependent inhibition of NTHi invasion of alveolar epithelial cells.

### Endocytosis of Surfactant Lipids Inhibits Signaling Pathways Involved in NTHi Internalization

NTHi was shown to enter bronchial epithelial cells through macropinocytosis (50). NTHi internalization seems to be mediated by either direct bacterium binding to integrins or indirect binding through extracellular matrix proteins (51, 52). Integrin signaling leads to phosphorylation of focal adhesion kinase (FAK), followed by activation of Rac1 GTPase and PI3K to induce actin polymerization and membrane protrusion (52). We previously showed that activation of both Rac1 and PI3K is essential for NTHi entry in A549 epithelial cells (7).

**Figure 7:**
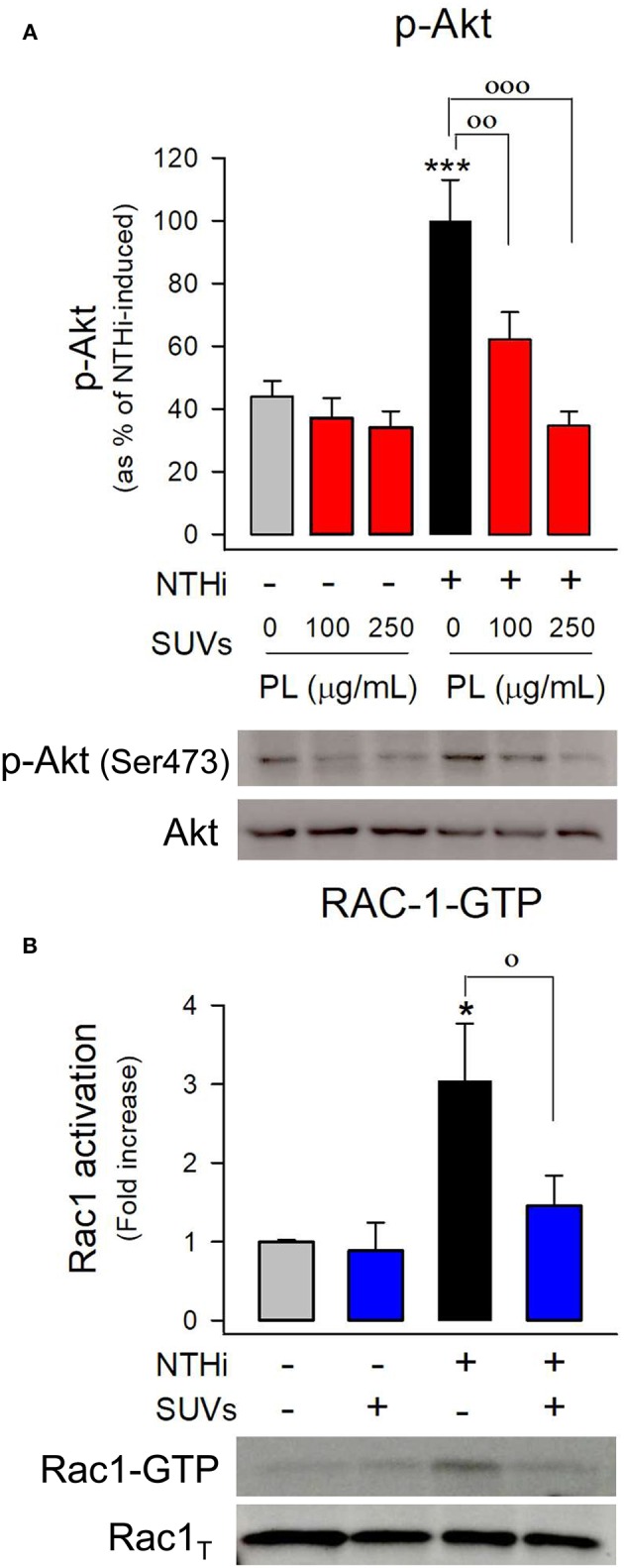
Endocytosis of surfactant lipids inhibits signaling pathways involved in NTHi internalization. **(A)** Mouse pneumocytes were preincubated with SUVs of surfactant lipids (100 or 250 μg PL/ml) for 24 h. Then cells were washed and infected with otitis NTHi for 45 min. Cells were lysed, and p-Akt and total Akt were analyzed by western blot. p-Akt was normalized to total Akt, and data are shown as percentage of NTHi-induced Akt phosphorylation in the absence of lipids. Mean ± SEM of three independent experiments performed in triplicate are shown. ANOVA followed by Bonferroni multiple-comparison test was used. ^***^p < 0.001 when compared with untreated and uninfected cells. ^◦◦^ *p* < 0.01 and ^◦◦◦^*p* < 0.001 when compared with infected pneumocytes in the absence of surfactant lipid vesicles. **(B)** MLE-12 cells were preincubated with SUVs of surfactant lipids (250 μg PL /ml) for 24 h. Then cells were infected with otitis NTHi for 2 h and activation of the GTPase Rac1 was analyzed by pull-down and western blot. Rac1-GTP and total Rac1 were quantified, and data are shown as percentage of NTHi-induced Rac1 activation (fold increase) relative to uninfected cells in the absence of lipids. Mean ± SEM of four independent experiments performed in triplicate are shown. ANOVA followed by the Bonferroni multiple-comparison test was used. ^*^p < 0.05 when compared with untreated and uninfected cells. ◦*p* < 0.05 when compared with infected pneumocytes in the absence of surfactant lipids.

To determine whether endocytosed surfactant vesicles reduced bacterial entry into pneumocytes by blocking key signaling pathways required for NTHi internalization, the next step was to evaluate NTHi-induced PI3K-Akt and Rac1 activation in mouse and human pneumocytes preincubated with or without surfactant lipid vesicles (Figure 7 and Supplementary Figure 4). We found that NTHi infection induced Akt phosphorylation (Figure 7A) and Rac1 GTPase activation (Figure 7B) in mouse pneumocytes, as previously described in human A549 cells (7). Endocytosed surfactant lipid vesicles inhibited NTHi-induced Akt phosphorylation in mouse (Figure 7A) and human (Supplementary Figure 4) pneumocytes. Furthermore, endocytosed surfactant lipids blocked NTHi-induced Rac1 activation (Figure 7B).

These results suggest that endocytosis of surfactant phospholipid vesicles interferes with cytoskeletal reorganization required for membrane protrusions that facilitate bacterial entry in pneumocytes.

### Surfactant Administration Protects From NTHi Infection *in vivo*

**Figure 8:**
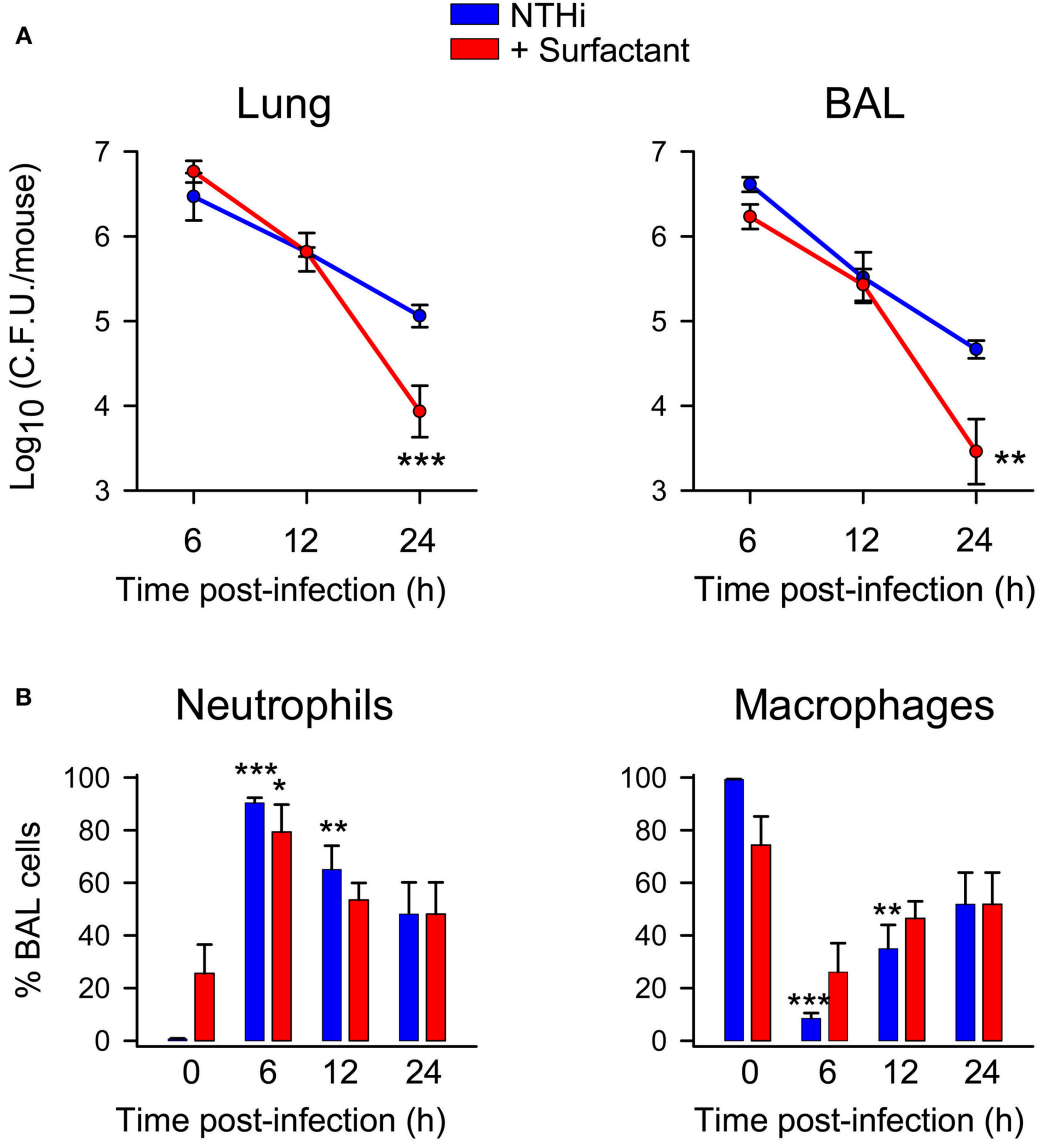
Surfactant treatment accelerated bacterial clearance in lung tissue and BAL of NTHi infected mice. Mice were intratracheally infected with otitis NTHi strain (4 × 10^7^ C.F.U./mouse), and the hydrophobic fraction of native pulmonary surfactant (25 mg PL/kg body weigh) was simultaneously instilled into the lungs (or the same volume of PBS in the untreated infected group). Mice were euthanized at 6, 12, or 24 h postinfection. Lungs were harvested, weighed, and homogenized (left lung) or used for BAL extraction (right lung) (*n* = 5 mice each infected group; *n* = 3 mice each uninfected group). **(A)** The numbers of viable bacteria in lung homogenates and BAL were assessed by colony counting and expressed as log_10_ (C.F.U./mouse). Results are mean ± SEM. ANOVA followed by Bonferroni multiple-comparison test was used. ^**^p < 0.01, and ^***^p < 0.001 when compared with untreated infected groups. **(B)** Percentage of alveolar macrophage and neutrophil counts in BAL. Results are mean ± SEM. ANOVA followed by the Bonferroni multiple-comparison test was used. ^*^p < 0.05, ^**^p < 0.01, and ^***^p < 0.001 when compared with the corresponding uninfected group.

Lastly, we wondered whether lung surfactant administration could protect from NTHi infection *in vivo*. To test this hypothesis, we infected CD1 mice intratracheally with a clinical NTHi strain from otitis patients, and simultaneously we instilled the hydrophobic fraction of native surfactant that contains surfactant lipids and the hydrophobic proteins SP-B and SP-C. Then, bacterial burden was assessed in whole lung tissue and BAL after 6, 12, and 24 h post infection. Figure 8 shows that administration of exogenous lung surfactant significantly diminished bacterial load in lung tissue and BAL of NTHi infected mice 12–24 h after surfactant administration, supporting the notion that the lipid component of lung surfactant protects against NTHi infection. Analyses of cell types in BAL from infected mice treated with or without surfactant revealed that recruited cells consisted predominantly of neutrophils (Figure 8). Neutrophil recruitment was similar in infected mice treated with surfactant compared with untreated infected animals. Control experiments indicate that neutrophil recruitment did not significantly increase in uninfected mice treated with surfactant compared with untreated uninfected mice (Figure 8). These results suggest that the accelerated clearance of NTHi in mice treated with surfactant was not associated with increased neutrophil infiltration at 6, 12, and 24 h post-infection in surfactant-treated mice compared with untreated mice.

## Discussion

Lung surfactant has two essential functions: keeping the alveolus open and host defense. These functions are inseparably coordinated and depend on the complexity of surfactant's components (19). Whereas surfactant proteins SP-A and SP-D have a clear role in lung defense, attention to surfactant lipids and hydrophobic surfactant proteins has been focused on surface tension properties. Whether surfactant lipids participate in the control of infection by inhaled pathogens remains mostly unaddressed.

In the present study, we demonstrated that the lipid component of pulmonary surfactant interferes with pathogen-host interaction between NTHi and pneumocytes through two different mechanisms. On the one hand, large extracellular surfactant vesicles bind to NTHi and function as biological barriers to reduce the number of bacteria adhered and internalized in alveolar epithelial cells. On the other hand, small surfactant vesicles were rapidly endocytosed by pneumocytes, mainly via the scavenger receptor SR-BI that mediates clathrin- and dynamin-independent endocytosis (53). The endocytosed surfactant lipids inhibited NTHi invasion by blocking bacterial uptake in pneumocytes.

Macropinocytosis is exploited by several intracellular pathogens as a means of entry into cells (54). This process is responsible for the uptake of large particles, >0.5 μm in diameter. The NTHi entry into pneumocytes requires direct or indirect bacterial binding to integrins, which initiate the activation of the Src/GEF Vav2/Rac1 GTPase/Pak1 signaling axis for polymerization of microtubules and cell ruffling (6, 7). These cytoskeletal remodeling proteins are bound to phosphatidylinositol (PtdIns)(4, 5)P2, which is enriched at the macropinocytic site in early stages of macropinosome development (54–56). Interestingly, PtdIns(4, 5)P2 disappearance is critical for macropinosome formation. At this stage, PtdIns(3,4,5)P3 is formed by PI3K activity. PtdIns(3,4,5)P3 is essential for cup closure (54–56). By recruiting adaptors, GEFs and GAPs that contain PtdIns(3,4,5)P3-interacting PH domains, the cell is able to dictate the activity of Rho family GTPases that direct the polymerization of actin required for macropinosome closure. Consistent with this, we previously found that inhibition of PI3K or Rac1 activation abrogates NTHi internalization (6, 7).

In this study, we show that NTHi-induced Rac1 GTPase activation and Akt phosphorylation by pneumocytes was inhibited by endocytosed surfactant lipids. Therefore, small surfactant lipid vesicles, once endocytosed, block intracellular signaling pathways that are required for NTHi entry into pneumocytes. The mechanism by which endocytosed lipid vesicles show such inhibitory effects is unknown. Given that clathrin-independent endocytosis of small particles also requires PtdIns(4,5)P2 for membrane curvature and invagination (55, 56) as well as Src, Rac1, or ARF signaling proteins that bind to PtdIns(4,5)P2, it is possible that endocytosis of lipid vesicles could shuttle PtdIns(4,5)P2 and associated proteins from the plasma membrane into internal membranes. The sequestration of PtdIns(4,5)P2 and Src, Rac1, or ARF could inhibit bacterial entry since depletion of PtdIns(4,5)P2 in the macropinocytic site reduces the macropinocytic or phagocytic efficiency (57).

To assess the beneficial role of lung surfactant lipids during NTHi infection, we performed *in vivo* experiments treating NTHi-infected mice with the hydrophobic fraction of native surfactant isolated from rat lungs. We found that this surfactant fraction, composed of PL, SP-B, and SP-C, was able to bind to NTHi, which led to a significant reduction of NTHi capability to self-aggregate. NTHi aggregation is responsible for microcolony formation on the epithelial surfaces, facilitating bacterial colonization of the airways (58). Administration of the hydrophobic fraction of native surfactant to NTHi infected mice significantly accelerated bacterial clearance in lung tissue and BAL. Surfactant treatment did not significantly affect early neutrophil recruitment in infected mice, suggesting that surfactant vesicles are directly involved in preventing bacterial adhesion to the epithelium and facilitating the actions of airway macrophages, neutrophils, antimicrobial proteins, and mucociliary clearance in promoting bacterial clearance.

Surfactant phospholipid concentration has been reported to decrease in COPD and idiopathic pulmonary fibrosis (30, 59). Smokers have reduced surfactant phospholipid levels, as well as impaired biophysical activity (30, 31). In asthma or pneumonia, reduced levels of phosphatidylcholine and changes in phospholipid composition have also been found (30). Surfactant lipids are also significantly altered in the aged lung in both mice and humans (60). In addition, pathological conditions, such as previous infection with rhinovirus, can reduce the inflammatory response and cause delay in NTHi clearance (61). Thus, in a context of immune dysregulation, the protective effect of pulmonary surfactant on NTHi respiratory infection may be more necessary. Therapeutic interventions with the hydrophobic fraction of surfactant might be of potential benefit in respiratory diseases with NTHi infection because exogenous surfactant protects against NTHi and improves lung biophysical function. In addition, the use of small surfactant-like vesicles as vehicles for inhaled drug administration may have further added value. This lipid-based host-directed strategy would offer novel prophylactic or therapeutic options against chronic, recurrent, or drug-resistant respiratory infections.

## Author Contributions

BG-F conceived, designed, and performed all the experiments, collected, analyzed, and interpreted all data, and wrote the manuscript. ZG-C, CM, AI-C, and LdT performed research. AdL, BE, and JG designed and performed *in vivo* experiments. SC-L contributed tools, and provided expertise. CC conceived the study, designed the experiments, interpreted and organized all data, and wrote the manuscript.

### Conflict of Interest Statement

The authors declare that the research was conducted in the absence of any commercial or financial relationships that could be construed as a potential conflict of interest.
