# Supplementary material for: Lung Surfactant Lipids Provide Immune Protection Against Haemophilus influenzae Respiratory Infection
Source: Front Immunol. 2019 Mar 18;10:458. doi: 10.3389/fimmu.2019.00458 (PMC6431623; doi:10.3389/fimmu.2019.00458)
Supplement: Supplementary file 1 [file Data_Sheet_1.pdf]

frontiers

Supplementary Material

Lung surfactant lipids provide immune protection against *Haemophilus influenzae* respiratory infection

Belen García-Fojeda[1,2], Zoe González[1,2], Alba de Lorenzo[1,2], Carlos Minutti[1,2], Lidia de Tapia[1], Begoña Euba[2,3], Alba Iglesias-Ceacero[1], Sonia Castillo Lluva[1], Junkal Garmendia[2,3], Cristina Casals[1,2]*

[1] Department of Biochemistry and Molecular Biology I, Complutense University of Madrid, 28040 Madrid, Spain.

[2] Centro de Investigación Biomédica en Red de Enfermedades Respiratorias (CIBERES), Instituto de Salud Carlos III, 28029 Madrid, Spain.

[3] Instituto de Agrobiotecnología, CSIC, Mutilva, 31192 Navarra, Spain.

***Correspondence:**
Cristina Casals, *Department of Biochemistry and Molecular Biology I, Faculty of Biology, Complutense University of Madrid, 28040 Madrid, Spain, Tel.: (34) 91394426. † e-mail: ccasalsc@ucm.es*

Keywords: nontypeable *Haemophilus influenzae*, pulmonary surfactant, phospholipids, alveolar epithelial cells, host-pathogen interaction, bacterial invasion, RAC-1, PI3K/Akt.

Running title: **Surfactant lipids limit NTHi infection**

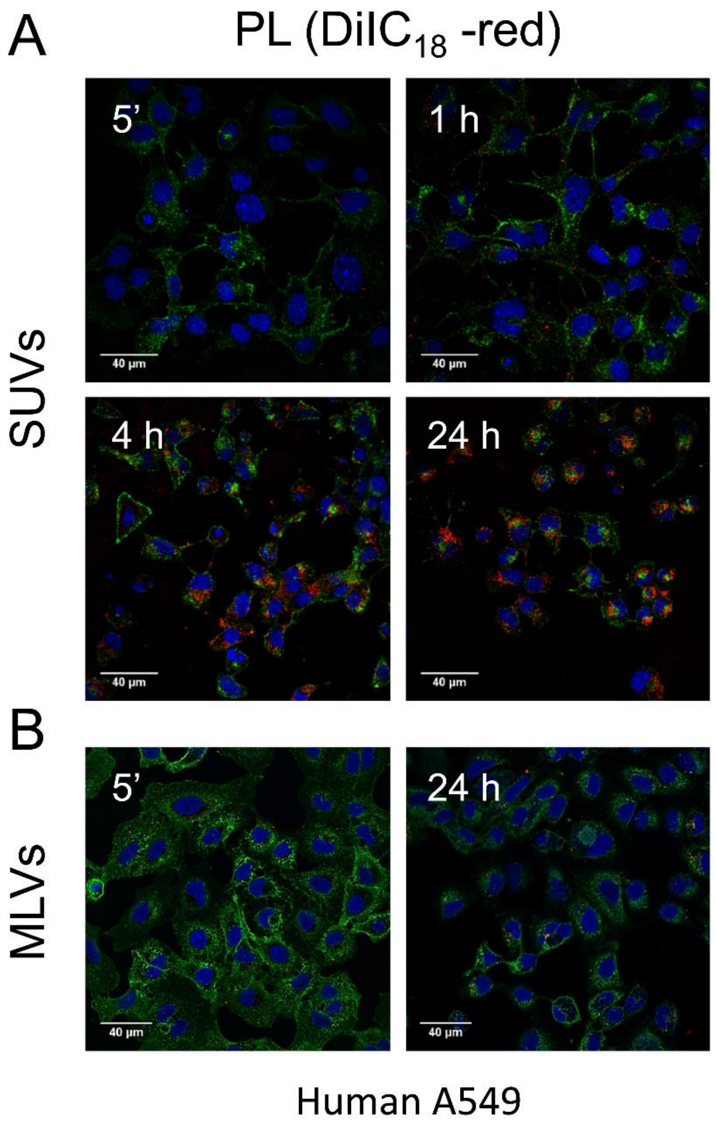

Supplementary Figure 1. Small vesicles, but not multilamellar vesicles, of pulmonary surfactant are endocytosed by human pneumocytes. A549 pneumocytes were incubated with DiIC18(3)-labelled SUVs (**A**) or MLVs (**B**) composed of a mixture of surfactant lipids (250 µg PL/ml) for the indicated time. Then cells were washed, fixed, and stained with Alexa Fluor 488-conjugated WGA and DAPI, and analyzed by confocal microscopy. Representative micrographs are shown.

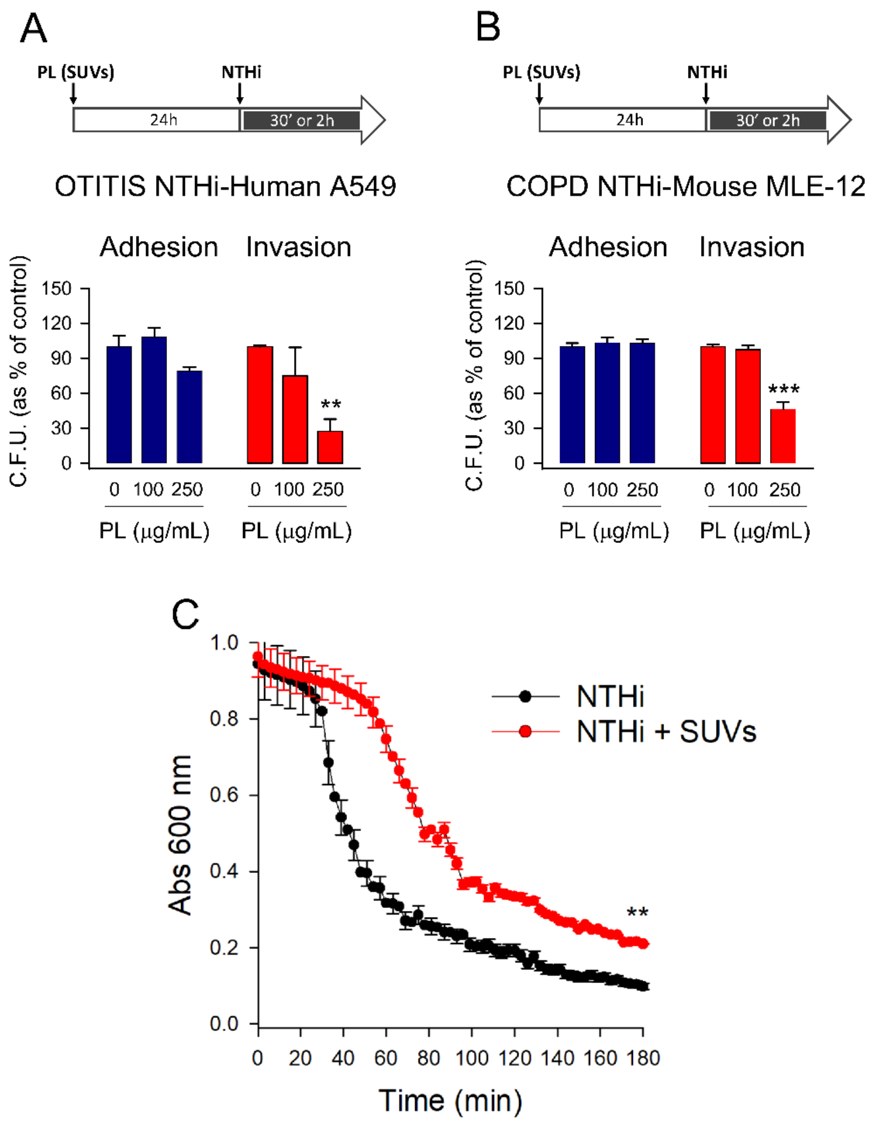

Supplementary Figure 2. Endocytosed surfactant lipids inhibit invasion of NTHi clinical strains in pneumocytes. Human A549 (**A**) or mouse MLE-12 (**B**) cells were pre-incubated with SUVs (100 and 250 µg PL/ml) composed of a mixture of surfactant lipids for 24 h. Then cells were infected with NTHi clinical strains from patients with otitis media (**A**) and COPD (**B**), and adhesion and invasion experiments were performed. For adhesion experiments, cells were infected for 30 min, washed, and lysed. For invasion assays, cells were infected for 2 h, washed, and incubated for an additional 1 h with gentamicin to kill extracellular bacteria. The resulting lysates were plated on sBHI agar. Data are shown as percentage of C.F.U. relative to infected cells in the absence of lipids. Results are mean ± SEM of three independent experiments performed in triplicate. ANOVA followed by the Bonferroni multiple-comparison test was used. **p < 0.01, and ***p < 0.001 when compared with infected pneumocytes in the absence of lipids. **(C) SUVs of surfactant lipids bind to NTHi and decrease bacterial self-aggregation**. NTHi bacteria (otitis strain) were incubated in the presence or absence of SUVs (250 µg PL/ml). Bacterial aggregation was monitored by measuring the decrease of absorbance at 600 nm every three minutes. Results are mean ± SEM of two independent experiments. Student's t-test was used. **p < 0.01.

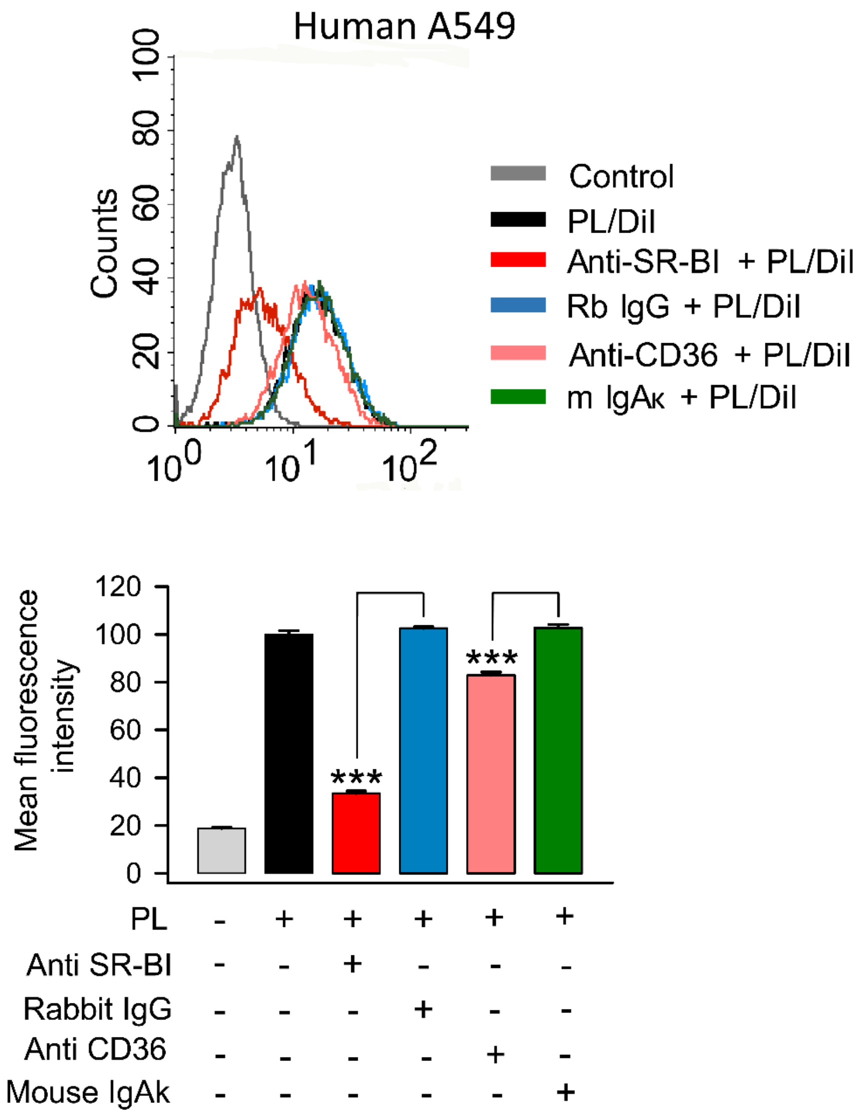

Supplementary Figure 3. SR-BI and CD36 mediate endocytosis of small surfactant vesicles in human pneumocytes. Human epithelial cells were incubated for 30 minutes with either anti-SR-BI, anti-CD36 blocking antibodies, or their respective controls, rabbit IgG or mouse IgAκ. Then cells were incubated with $DiIC_{18}(3)$-labelled SUVs (100 μg/ml) for an additional 60 min. Cells were fixed and PL uptake analyzed by flow cytometry. The mean fluorescence intensity is represented as percentage of positive control, which is cells incubated with $DiIC_{18}(3)$-labelled SUVs in the absence of antibodies. Data are presented as mean ± SEM of two independent experiments run in triplicate. ANOVA followed by the Bonferroni multiple-comparison test was used. ***$p < 0.001$.

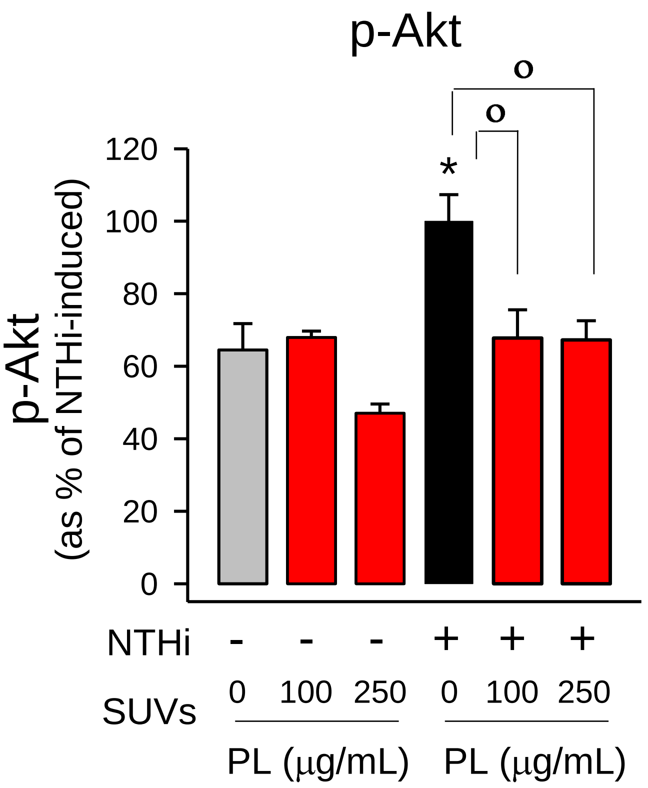

Supplementary Figure 4. Endocytosed surfactant lipids inhibit NTHi-induced Akt phosphorylation in human pneumocytes. A549 pneumocytes were incubated with 100 or 250 μg/ml of surfactant lipids (SUVs) for 24 h. Then, cells were washed and infected with otitis NTHi for 45 minutes, and p-Akt and total Akt were analyzed by western blot as explained in the Materials and Methods section. Data are expressed as percentage of NTHi-induced Akt phosphorylation in the absence of lipids. Mean ± SEM of three independent experiments performed in triplicate are shown. ANOVA followed by the Bonferroni multiple-comparison test was used. *$p < 0.05$ when compared with untreated uninfected cells. $^{o}p < 0.05$ when compared with infected pneumocytes in the absence of surfactant lipid vesicles.
